# The Interaction between Reactive Peritoneal Mesothelial Cells and Tumor Cells via Extracellular Vesicles Facilitates Colorectal Cancer Dissemination

**DOI:** 10.3390/cancers13102505

**Published:** 2021-05-20

**Authors:** Simona Serratì, Letizia Porcelli, Francesco Fragassi, Marianna Garofoli, Roberta Di Fonte, Livia Fucci, Rosa Maria Iacobazzi, Antonio Palazzo, Francesca Margheri, Grazia Cristiani, Anna Albano, Raffaele De Luca, Donato Francesco Altomare, Michele Simone, Amalia Azzariti

**Affiliations:** 1 Laboratory of Nanotechnology, IRCCS Istituto Tumori Giovanni Paolo II, 70124 Bari, Italy; s.serrati@oncologico.bari.it (S.S.); a.palazzo@oncologico.bari.it (A.P.); 2 Laboratory of Experimental Pharmacology, IRCCS Istituto Tumori Giovanni Paolo II, Viale O. Flacco 65, 70124 Bari, Italy; l.porcelli@oncologico.bari.it (L.P.); m.garofoli@oncologico.bari.it (M.G.); r.difonte@oncologico.bari.it (R.D.F.); r.m.iacobazzi@oncologico.bari.it (R.M.I.); 3 Department of Surgery Oncology, IRCCS Istituto Tumori Giovanni Paolo II, 70124 Bari, Italy; f.fragassi@studenti.uniba.it (F.F.); raffaele.deluca@oncologico.bari.it (R.D.L.); donatofrancesco.altomare@uniba.it (D.F.A.); m.simone@oncologico.bari.it (M.S.); 4 Pathology Department, IRCCS Istituto Tumori Giovanni Paolo II, 70124 Bari, Italy; l.fucci@oncologico.bari.it (L.F.); g.cristiani@oncologico.bari.it (G.C.); 5 Department of Experimental and Clinical Biomedical Sciences, University of Florence, 50134 Florence, Italy; francesca.margheri@unifi.it; 6 Clinical Trial Center, IRCCS Istituto Tumori Giovanni Paolo II, 70124 Bari, Italy; a.albano@oncologico.bari.it; 7 Department of Emergency and Organ Transplantation, University Aldo Moro of Bari, 70124 Bari, Italy

**Keywords:** colorectal cancer, peritoneal carcinomatosis, CD44, mesothelial cells, MMT

## Abstract

**Simple Summary:**

Emerging evidence has suggested that cancer-derived extracellular vesicles (EVs) have a crucial role in mediating directional metastasis to the peritoneal surface in colorectal cancer (CRC). We investigated the EV-mediated crosstalk between tumor and mesothelial cells which may drive remodeling of the premetastatic niche to allow tumor spread to the peritoneal surface. Our findings demonstrated that cancer-derived EVs triggered apoptosis and reduced mesothelial cell invasiveness and mesothelial-to-mesenchymal transition. On the other hand, mesothelial cells actively supported tumor invasion by releasing EVs, which induced upregulation of the major pro-invasive system in tumor cells. For the first time, we provide evidence of EV-driven mechanisms of CRC progression in patient-derived models, highlighting the crucial role of EVs in the reprogramming of mesothelial and tumor cells to establish the metastatic process.

**Abstract:**

Advanced colorectal cancer (CRC) is highly metastatic and often results in peritoneal dissemination. The extracellular vesicles (EVs) released by cancer cells in the microenvironment are important mediators of tumor metastasis. We investigated the contribution of EV-mediated interaction between peritoneal mesothelial cells (MCs) and CRC cells in generating a pro-metastatic environment in the peritoneal cavity. Peritoneal MCs isolated from peritoneal lavage fluids displayed high CD44 expression, substantial mesothelial-to-mesenchymal transition (MMT) and released EVs that both directed tumor invasion and caused reprogramming of secretory profiles by increasing TGF-β1 and uPA/uPAR expression and MMP-2/9 activation in tumor cells. Notably, the EVs released by tumor cells induced apoptosis by activating caspase-3, peritoneal MC senescence, and MMT, thereby augmenting the tumor-promoting potential of these cells in the peritoneal cavity. By using pantoprazole, we reduced the biogenesis of EVs and their pro-tumor functions. In conclusion, our findings provided evidence of underlying mechanisms of CRC dissemination driven by the interaction of peritoneal MCs and tumor cells via the EVs released in the peritoneal cavity, which may have important implications for the clinical management of patients.

## 1. Introduction

Colorectal cancer (CRC) is currently the third cause of cancer-related deaths in the world. About 25% of CRC patients develop peritoneal carcinomatosis (PC) and intraperitoneal metastasis is present in 80% of patients who succumb to CRC [1,2]. Hence, there is an urgent need to characterize the mechanisms through which peritoneal metastasis occurs in the early stages of CRC and identify associated biomarkers to improve the diagnosis and treatment of CRC patients. For many years, scientists have speculated on the process of tumor development as the result of the accumulation of a significant number of oncogenic mutations [3,4]. In 1889, S. Paget proposed a theory in which the metastatic homing of tumor cells was governed by interaction between the *seed* (metastatically competent cancer cells) and the *soil* (the permissive microenvironment of specific organs) [5]. In this perspective, increasing evidence, especially regarding epithelial ovarian cancer and gastric cancer, has demonstrated the pivotal role of exosomes and of extracellular vesicles (EVs) in governing the bidirectional crosstalk between cancer cells and the host tissue to generate a premetastatic environment in the peritoneal cavity [6,7,8,9]. The mechanisms of peritoneal dissemination in CRC have only very recently been uncovered and the role of exosomes in host–tumor cell communication is almost unknown [1]. In ovarian cancer, peritoneal mesothelial cells (MCs) have been demonstrated to actively contribute to metastasis by regulating proliferation, invasion, and adhesion of tumor cells to the peritoneal mesothelium [6,10], while in CRC, peritoneal MCs are believed to protect against metastasis [5,11]. However, recent findings have shown that the peritoneal MCs are affected by tumor mediators released in the peritoneal cavity and switch from their barrier function to acquire features which contribute to metastasis both in CRC and ovarian cancer [12,13]. Among tumor-induced transformations, TGF-β-dependent activation of mesothelial-to-mesenchymal transition (MMT) in peritoneal MCs changes adherent cells into floating cells with an activated fibroblast phenotype [9,14]. This event has been demonstrated to facilitate access of CRC metastasis into the peritoneal cavity [15]. Intraperitoneal CRC progression is also stimulated by senescent MCs [16]. It has been evidenced that senescent peritoneal MCs display an increased expression of adhesion molecules, such as CD43 and ICAM-1, which enhance adherence of tumor cells to MCs, thus favoring peritoneal invasion [12,17]. Recently, Nakamura et al. have reported that tumor-derived exosomes promote ovarian cancer cell invasion through transfer of CD44 to peritoneal MCs, highlighting the crucial role of CD44 expression in the acquisition of a mesenchymal phenotype in mesothelial cells and in the interaction of the latter with tumor cells [18]. CD44 is a multifunctional cell surface adhesion receptor [19] whose expression is correlated to poor prognosis in CRC [20]. One of the functions of CD44 is to regulate cell migration by inducing epithelial-to-mesenchymal transition (EMT) and the release of extracellular matrix (ECM) remodeling factors such as TGF-β1 and matrix metalloproteinases (MMPs) from tumor cells, thereby promoting the progression of diverse tumors, including CRC [20,21]. Of note, CD44 expression has been described as a distinctive biomarker of reactive mesothelial cells in peritoneal washings that may contribute to the staging protocols for both ovarian and endometrial carcinomas [22]. On the basis of this background, we hypothesized that reactive mesothelial cells are present in the peritoneal lavage fluids (PLFs) of CRC patients and release EVs that facilitate crosstalk with tumor cells to promote metastasis in the peritoneal cavity. Hence, we investigated the biological effects of EV-mediated crosstalk between CRC cell lines and mesothelial cells isolated from PLFs collected during surgery to explore the tumor-supportive effect of such crosstalk and provide evidence that peritoneal fluid EVs play a role in the pathophysiological changes that occur in the peritoneum, ultimately leading to peritoneal carcinomatosis.

## 2. Materials and Methods

Primary and secondary antibodies as well as other materials used in this study are reported in Table S1.

### 2.1. Cell Lines

The human colorectal adenocarcinoma cells lines COLO 205 (ATCC CCL-222, LGC Standards S.r.l., Sesto San Giovanni, Italy) and Caco-2 (ATCC HTB-37, LGC Standards S.r.l., Italy) were cultured according to the manufacturer’s instructions and supplemented with 10% exosome-depleted fetal bovine serum (FBS South America, exosome depleted, Bio West, Nuaillé, France). dFB (Normal Human Dermal Fibroblasts, Lonza, Basel, Switzerland) were grown in FGM-2 Bullet Kit, Lonza, Switzerland, supplemented with 10% exosome-depleted FBS. The cells were utilized for the experiments after routine testing for mycoplasma contamination.

### 2.2. Peritoneal Lavage Fluid (PLF) Collection

The study was approved by the local Ethical Committee (document no. 716/2019 CE). A series of twelve colon cancer patients were selected by our clinician. All the patients signed written informed consent for the investigations and underwent surgery between April 2019 and February 2020 at the Surgery Unit in our Institute. Peritoneal washings were performed immediately after abdominal incision or laparoscopy and before tumor manipulation. Then, 500 mL of isotonic saline solution were instilled in the abdominal cavity above the tumor site and kept in contact with the tissues for 3 min, after which 50 mL of solution (PLF) was collected and immediately processed. The PLFs were centrifuged at 1300 rpm for 10 min at RT; the supernatant and the pellet were used for the isolation of EVs and mesothelial cell lines, respectively.

### 2.3. Primary Culture of Peritoneal Carcinomatosis Cells (PC1-V)

A 42 -year-old male patient diagnosed with primary colorectal cancer (TNM stage: T4) presented with malignant ascites and peritoneal seeding. The patient was extensively informed about the investigations and provided written consent. The study was approved by the local Ethical Committee (document no. 656/17 CE). Malignant ascites was collected during primary debulking surgery. Tumor cells were isolated and cultured in Dulbecco’s modified Eagle’s medium (DMEM, Euroclone, Pero, Italy) supplemented with 2 mM glutamine (Euroclone, Italy), 100 UI/mL penicillin, 100 µg/mL streptomycin (Euroclone, Italy), 10% of FBS exosome-depleted (EXO-FBS-250A-1, System Biosciences, Abingdon, UK), at 37 °C in a humidified atmosphere containing 5% CO_2_. These cells were named PC1-V.

### 2.4. Primary Cultures of Human Peritoneal Mesothelial Cells

The 12 PLFs were centrifuged at 1300 rpm for 10 min at RT and the pellet was then used to isolate the human peritoneal MCs that were named MC1-12. To enrich the portion of human peritoneal MCs, fibroblasts were removed through immunomagnetic depletion by using anti-Fibroblast MicroBeads (MiltenyiBiotec, Bergisch Gladbach, Germany) according to the manufacturer’s instructions. The negative cells were plated in Dulbecco’s modified Eagle’s medium (DMEM, Euroclone, Italy) supplemented with 2 mM glutamine (Euro-clone, Italy), 100 UI/mL penicillin, 100 µg/mL streptomycin (Euroclone, Italy), 10% of FBS exosome-depleted (EXO-FBS-250A-1, System Biosciences, Abingdon, UK), at 37 °C in a humidified atmosphere containing 5% CO_2_. All the experiments were performed utilizing MC1-12 from passages 2 to 6.

### 2.5. EV Isolation

Extracellular vesicles (EVs) were isolated from the 12 PLFs and from the conditioned medium (CM) collected from the MCs and tumor cell lines. The EVs from the PLFs were obtained from 50 mL of solution. For the culture media, EVs were isolated from CM after it had been in contact with cells for 24 h at a confluency of the cells of 70–80%. Then, 30 mL of CM or 50 mL of PLF were centrifuged for 15 min at 3000× *g* and the supernatants were filtered using 200-nm pore size filters to eliminate larger extracellular vesicles. The resulting cell-free liquid was ultracentrifuged at 100,000× *g* for 70 min. The pellet was washed with sterile PBS, centrifuged again at 110,000× *g* for 70 min, and then resuspended in PBS [23]. The pooled EVs were stored at −80 °C. EV concentration was determined by protein quantification (Bradford Protein Assay; Bio-Rad Laboratories, Hercules, CA, USA) according to the published literature [24]. The EVs protein yield from 50 mL of PLF, respunsended in 300 μL PBS, was in a range between 0.58 ± 0.12 and 1.43 ± 0.52 μg/μL. The EV isolation details are reported in Table S2. An amount of EVs corresponding to 200 µg/mL of protein concentration was used in all the experiments.

### 2.6. Nanoparticle Tracking Analysis (NTA)

The EVs were analyzed using the NanoSight NS300 (Malvern Panalytical, Malvern. UK) according to the manufacturer’s instructions (NanoSight NS300 User Manual, MAN0541-02-EN, 2018) [25]. Further, 5–10 µL of each EV sample were diluted resulting in a particle per frame rate of between 10 and 50. All the samples were analyzed in triplicate; 30 s video images were acquired and analyzed by the NanoSight NTA 3.2 software.

### 2.7. EV Characterization by Flow Cytometry (FCM)

FCM instrument preparation and setup was as reported in the supplementary file M&M-S1 of [26]. Each EV sample was preincubated with 5 µL of Super Bright Complete Staining Buffer to prevent nonspecific polymer interactions for 30 min in the dark at 2−8 °C. Then the EVs (never permeabilized prior to the addition of the antibodies) were labeled with anti-human antibodies and stored for 30 min in the dark at 2−8 °C. After staining, the EVs were collected and analyzed with an Attune^TM^NxT Acoustic Focusing Cytometer (ThermoFisher, Waltham, MA, USA) equipped with four lasers (405 nm violet, 488 nm blue, 561 nm yellow, and 637 nm red). The final data were analyzed using the Attune^TM^NxT Analysis Software (ThermoFisher) [26].

### 2.8. PKH67 Labeling of EVs and Uptake into Recipient Cells

The EVs were labeled using PKH67 Fluorescent Cell Linker kits according to the manufacturer’s instructions. To examine the uptake of EVs into recipient cells, the cells were placed onto 6-well plate coverslips at a density of 1 × 10^4^ cells per well. After 24 h, the cells were washed three times in PBS and the culture medium containing PKH67-labeled EVs solution was added to each well. Cells were cultured for 6 h at 37 °C in a humidified atmosphere with 5% CO_2_ and then fixed with 3.7% paraformaldehyde solution according to the standardized protocol [27].

### 2.9. Cell Morphological Analysis

Freshly isolated cells were observed daily under phase microscope (OLYMPUS CKX41, Hamburg, Germany). The documented images are representative of the morphological characteristics of the mesothelial and tumor cells included in the study.

### 2.10. Immunohistochemistry (IHC)

The 10^4^ cells were grown for 24 h on Lab-Tek Chamber Slides (Nunc, Waltham, MA, USA). The protocol was carried out as previously described [28]. See Table S1 for the antibodies used. Ten million PC1-V cells were fixed in 10% buffered neutral formalin for about 3 h. Subsequently, a paraffin cell block was obtained and sections of 3 microns were stained with hematoxylin eosin and immunostained for CDX2 and CK20 expression assessment. The specimens were compared with the original neoplastic cells found in the ascitic fluid. Evaluations were made directly by the pathologist.

### 2.11. Immunofluorescence (IF)

The immunofluorescences assays were performed as described previously [29]. Further, 2 × 10^3^ cells were grown on chamber slides as described above and incubated overnight with primary antibody. The slides were then incubated with appropriate fluorescent Alexa dye-labeled secondary antibody in PBS and counter-stained with 4′,6-diamidino-2-phenylindole (DAPI) nuclear stain (10 µg/mL) (Invitrogen/ThermoFisher Scientific, Waltham, MA, USA). For the immunofluorescence evaluation of mesothelial-to-mesenchymal transition markers, fixed mesothelial cells were stained with anti-α-SMA, anti-Vimentin, and Phalloidin-iFluor 555 was applied to the cells to visualize the arrangement of the actin cytoskeleton. Nuclei were stained with DAPI. The cells were observed under a fluorescence microscope (Leyca DMi8, Leyca Microsystem Imaging Solutions Ltd., Cambridge, UK) fitted with a digital camera.

### 2.12. Confocal Microscopy

The 2 × 10^3^ cells were incubated for 6 h with the culture medium containing PKH67-labeled EVs solution. The cells were then fixed and stained with anti-LAMP-1. The immunofluorescence assay was performed as described previously [30].

### 2.13. Western Blot

The protocol was carried out as described previously [24]. In particular, 30 µg of EVs protein lysate, or 50–80 µg of cell protein lysate, were run on a 4–20% Mini-PROTEAN^®^ TGX Stain-Free™ Gel (Bio-Rad Laboratories). The protein was transferred to a nitrocellulose membrane (Trans-Blot® Turbo™ Mini Nitrocellulose, Bio-Rad Laboratories) using the Trans-Blot® Turbo™ Transfer System (Bio-Rad Laboratories). The membrane was blocked and then incubated overnight at 4 °C with primary antibodies. On the following day, the membranes were incubated with the secondary antibody. Blot detection was performed with ChemiDoc™ Imaging Systems. See Table S1 for the antibodies used.

### 2.14. Cell Characterization by FCM

The 5 × 10^5^ cells were collected and incubated with fluorescent-tagged monoclonal antibodies and stored for 30 min in the dark at 2−8 °C. After staining, the cells were washed twice and analyzed using an Attune^TM^NxT Acoustic Focusing Cytometer (ThermoFisher), and the final data were analyzed using the Attune^TM^NxT Analysis Software (ThermoFisher).

### 2.15. Cell Apoptosis Assay

The FITC Annexin V Apoptosis Detection Kit II (BD Pharmingen^TM^–BD Biosciences, Erembodegem, Belgium,) was used to detect the amount of apoptotic cells. Then, 1 × 10^5^ MCs were treated for 48 h with 200 µg/mL of EVs. The cells were processed according to the instructions provided by the manufacturer and data analysis was performed by using the Attune^TM^NxT Analysis Software (ThermoFisher).

### 2.16. Gelatin Zymography

Conditioned medium was collected from mesothelial cells and cancer cells and a gelatine zymogram was performed to evaluate gelatinase activity on the gelatin substrate. Aliquots of culture medium (40 µg protein) were subjected to SDS-PAGE (8%, containing 0.13% gelatin) under non-reducing conditions and were subsequently subjected to zymography as described earlier [31].

### 2.17. Preparation of Conditioned Medium With and Without EVs.

The cells were grown to 70–80% confluence, then washed twice with phosphate buffered saline, and incubated for 24 h in the presence of complete medium supplemented with 10% of fetal bovine serum exosome depleted. The culture supernatant was centrifuged at 2000× *g* for 10 min and used immediately [31]. To obtain the conditioned medium EVs-depleted, the supernatant was centrifuged at 2000× *g* for 10 min and then at 10.000× *g* for 30 min. The resulting cell-free medium was ultracentrifuged at 100,000 g for 70 min and used immediately.

### 2.18. Invasion Assay

Boyden chambers were used to evaluate spontaneous and stimulated invasion (chemoinvasion) through Matrigel-coated porous filters as described previously [32]. For spontaneous invasion, 50 µL of cell suspension (6 × 10^3^ mesothelial cells and 10 × 10^3^ cancer cells) were placed in the upper compartment of the Boyden chamber and migration allowed to occur for 6 h (mesothelial cells) or overnight (cancer cells). For assessment of chemoinvasion, EVs (200 µg/mL of EV proteins) were dissolved in culture medium and placed in the lower wells. The same experiments were performed in presence of CM with or without EVs in the lower compartment of the Boyden chamber. Migrated cells were stained and analyzed as previously described [32]. Adherent cells were counted using a light microscope (×200 magnification), (OLYMPUS CKX41).

### 2.19. Viability Studies

Mesothelial cells were seeded in 96-well culture plates at a density of 5000 cells/well. After 24 h, the culture medium was replaced with fresh medium (100 µL) with EVs (200 µg/mL of EV proteins) from colon cancer cells (treated samples) and without EVs (Ctrl). Then, cells were further incubated for 24 and 48 h and cell viability was assessed by MTT assay as previously described [33]. Results were expressed as percent variation with respect to control ±s.d.

### 2.20. Quantitative Reverse Transcription–Polymerase Chain Reaction (RT-PCR) Analysis

The 5 × 10^6^ cancer cells were treated with EVs (200 µg/mL) from MCs for 24 h. The mRNA expression of uPA, uPAR, PAI-1, MMP-2, MMP-9, and TGF-β1 was assayed using the SYBR Green assay as described previously [29]. The results of three independent experiments run in triplicate were expressed as fold changes according to the 2^−ΔΔCT^ method. Primer sequences are reported in Table 1.

### 2.21. Senescence Assay

For the senescence assay, the MCs were plated in 12-well plated at a density of 50 × 10^4^/well and the next day, 200 µg/mL EVs or 10 ng/mL TGF-β1 were applied to cells and the assay was performed as reported in [34]. Representative pictures showing SA-β-Gal-stained cells were recorded by using an OLYMPUS CKX41 microscope equipped with a CCD camera.

### 2.22. Statistics

Statistical significance was calculated using the Student–Newman–Keuls test and a two-way analysis of variance (ANOVA) followed by the Bonferroni post hoc tests (GraphPad Prism vers. 5). Data were indicated with * *p* < 0.05, ** *p* < 0.01, and *** *p* < 0.001 compared to relative control.

## 3. Results

### 3.1. MCs in the PLF of CRC Patients Show High CD44 Expression, Release CD44^+^ EVs and Display Relevant MMT Stage

Primary MC cell lines were isolated from the PLFs of 12 consecutive patients with advanced CRC (Table 2).

**Figure 1:**
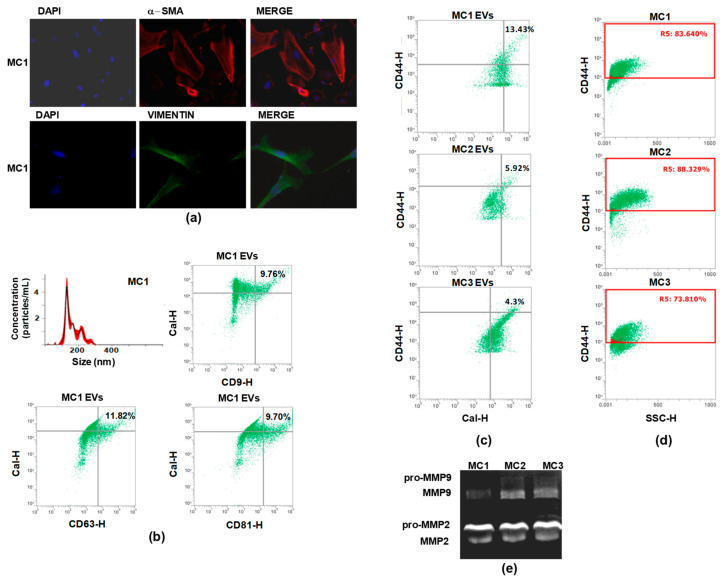
Characterization of mesothelial cells and of the EVs secreted in the conditioned medium (CM). (**a**) Representative picture showing IF evaluation of the basal expression of α-SMA (red) and vimentin (green) in MC1, MC2, and MC3. Nuclei are counterstained with Dapi (blue) (magnification ×100). (**b**) Nanoparticle tracking analyses showing the concentration and the size of EVs released by MC1. FCM dot plots showing the percentage of Cal^+^/CD9^+^, Cal^+^/CD63^+^, Cal^+^/CD81^+^ EVs populations released by MC1. (**c**) FCM dot plots showing the percentage of CD44^+^/Cal^+^ EVs released by MC1, MC2, and MC3. (**d**) Dot plots indicating the percentage of CD44 expression in MC1, MC2, and MC3. (**e**) Representative gelatin zymography showing that MC1, MC2, and MC3 secreted both pro-MMP-2/MMP-9 and active MMP-2/MMP-9.

Upon morphological examination, some cells still retained their cobblestone-like morphology while others displayed spindle cell morphology and lost cell–cell contact (Figure S1a). Accordingly, they expressed variable levels of calretinin, a discriminatory marker for mesothelial cells (Figure S1b,c, Figure S2a, and Table S3), as well as α-SMA and vimentin, revealing their dual epithelial–mesenchymal characteristics and heterogeneity of mesothelial cell types. Representative IF images of α-SMA and vimentin expression in the MC1 cell line are shown in Figure 1a.

The EVs released by mesothelial cells (MC EVs) were then characterized and CD44 expression analyzed. Preliminarily, the EVs collected from the conditioned medium (CM) of mesothelial cells were characterized by NTA and were found to range from 40 to 200 nm in size (size reported in Figure 1b). They stained positive for calretinin, specific biomarker of MCs, and at least one of the three tetraspanins CD9, CD63, and CD81, i.e., for exosomes and EV-selective biomarkers in general [35]. Representative dot plots showing positive staining for EV biomarkers are depicted in Figure 1b.

Next, we performed FCM analysis to assess CD44 expression on EV membranes. Data analysis showed that CD44 was present in 4–13% of the EV populations released by the twelve cell lines. Representative dot plots showing the percentage of CD44^+^ EVs are depicted in Figure 1c. We chose three cell lines, designated as MC1, MC2, and MC3, as representative models of the PLF MCs, and we assessed the membrane expression of the major homing cell adhesion molecule, CD44. FCM evaluation showed that CD44 membrane expression in MC1, MC2, and MC3 ranged between 70–80% of the cell populations (Figure 1d). The stage of MMT in MC1, MC2, and MC3 was determined by examining the activation level of MMP-2 and MMP-9 by gelatin zymography in the CM. The results of the assay showed that the three MC lines released both pro-MMP-2/MMP-9 and active MMP-2 and MMP-9 (Figure 1e, Figure S3a, and Table S4). Collectively, the data on MMP activation status, α-SMA expression, and CD44 expression highlighted that the cells displayed substantial MMT and an activated fibroblast phenotype [36].

### 3.2. MC EVs Are Successfully Internalized by Colon Cancer Cells to Activate Metastatic Signaling Pathways

**Figure 2:**
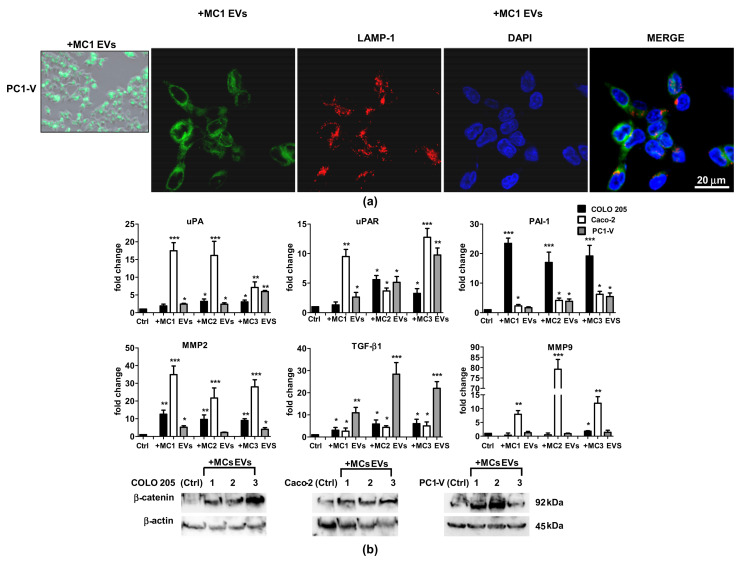
Uptake of MC EVs causes upregulation of metastatic signaling pathways in tumor cell lines. (**a**) Left side: Representative images showing the internalization of fluorescent PKH67- MC EVs (green) in the PC1-V cell line (magnification ×400). Right side: Representative images of fluorescence confocal microscopy showing PKH67-MC1 EV uptake and colocalization with LAMP-1 (red) in PC1-V cells (magnification ×1000). (**b**) Fold change of uPA, uPAR, PAI-1, MMP-2, MMP-9, and TGF-β1 transcript levels in cancer cells after treatment with MC1-3 EVs. Each value reported in the histograms is the mean ± SD of three different experiments performed in triplicate (* *p* < 0.05, ** *p* < 0.01, and *** *p* < 0.001 relative to each untreated control). (**c**) Representative immunoblots showing β-catenin protein expression in COLO 205, Caco-2, and PC1-V treated with MC1-3 EVs. β-actin is used as loading control.

The next step was to study the tumor-promoting activity of the MCs by evaluating the effect elicited by MC EVs on cancer cell motility. Preliminarily, we confirmed the uptake of MC EVs in the two commercial colon cancer cell lines, Caco-2 and COLO 205, and in the primary colon cancer cell line, PC1-V, established from the ascitic fluid of a 42-year-old patient with peritoneal carcinomatosis. The PC1-V cell line was characterized to confirm that it was representative of the tumor of origin. The PC1-V characterization data are shown in Figure S4. The MC EVs were then stained with PKH67 and uptake of the green fluorescent-EVs was evaluated by confocal fluorescence microscopy, confirming cell uptake and localization in lysosomes and endosomes, as demonstrated by the colocalization of PKH67-labeled EVs with lysosome marker LAMP-1 [30] (Figure 2a and Figure S5). We then investigated if the MC EVs were able to reprogram the cancer cells by increasing their invasive potential. We added 200 µg EVs isolated from the CM of MC1, MC2, and MC3 to COLO 205, Caco-2, and PC1-V cells to determine the mRNA expression level of ECM remodeling factors and β-catenin protein expression. We focused on the mRNA expression of MMP-2, MMP-9, urokinase plasminogen activator receptor (uPAR), urokinase plasminogen activator (uPA), and its inhibitor PAI-1 because their activation has been associated with peritoneal metastases in gastric cancer [37,38] and they are regulated by β-catenin as EMT-promoting effectors [20,39]. We also determined the mRNA expression of TGF-β1, which is one the factors supporting both tumor cell invasion and the paracrine induction of MMT in mesothelial cells [13]. The uptake of MC EVs by tumor cells caused a great increase in MMP-2 and MMP-9 mRNAs and a concomitant significant increase in uPA and uPAR, mainly in the Caco-2 cell line. Likewise, upon uptake of MC EVs, the mRNA levels of MMP2 and of uPA/uPAR increased in both the COLO 205 and PC1-V cell lines, albeit to a lesser extent than in the Caco-2 cell line. Interestingly, COLO-205 displayed the highest expression of PAI-1 mRNA. Activation of the invasive trait in CRC cell lines after the addition of MC EVs was further confirmed by the significant increase in TGF-β1 mRNA expression, particularly in the PC1-V cell line. All the data on mRNA expression analysis are shown in Figure 2b. β-catenin signaling activation was determined by Western blotting (Figure 2c, Figure S6a, and Table S3). β-catenin protein expression was shown to increase upon addition of MC EVs in all three colon cancer cell lines, further supporting the notion that the EVs triggered metastatic signaling pathways to activate in tumor cells.

### 3.3. MC EVs Trigger Effective Chemotactic Response in CRC Cell Lines

**Figure 3:**
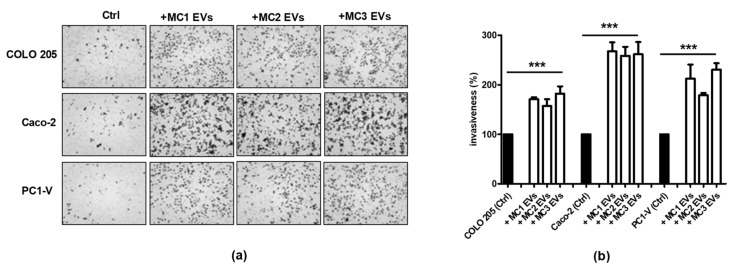
MC EVs release chemokinetic signals promoting tumor cells invasion. (**a**) Representative images showing that MC1-3 EVs promote cancer cell invasion (magnification ×100). (**b**) Quantification of cancer cell invasion percentage (*** *p* < 0.001 relative to control). Each value reported in the histograms is the mean ± SD of three different experiments performed in triplicate.

EVs can direct cell migration through the stroma by generating chemotactic gradients [40,41]. In order to investigate whether the EVs secreted by MC1, MC2, and MC3 also released chemotactic signals that could direct CRC cell line migration experiments of spontaneous migration (control condition) and stimulated invasion (chemoinvasion) through Matrigel-coated porous filters were performed. In chemoinvasion, the EVs were placed in the lower compartment of the well. Representative pictures are shown in Figure 3a. The three colon cancer cell lines displayed moderate invasive behavior per se, while Caco-2 cell line invasiveness increased by almost 3-fold and that of COLO 205 and PC1-V cell lines almost 2-fold versus control cells in response to chemotactic signals released by MC EVs (quantifications are reported in Figure 3b). These findings showed that the MCs we utilized in our experiments were chemotaxis-competent cells releasing EVs which transmitted chemokinetic signals and, once internalized by the tumor cells, were able to activate metastatic signaling pathways favoring ECM remodeling and tumor motility.

### 3.4. Tumor EVs Facilitate Clearance of Mesothelial Cells by Inducing Apoptosis

**Figure 4:**
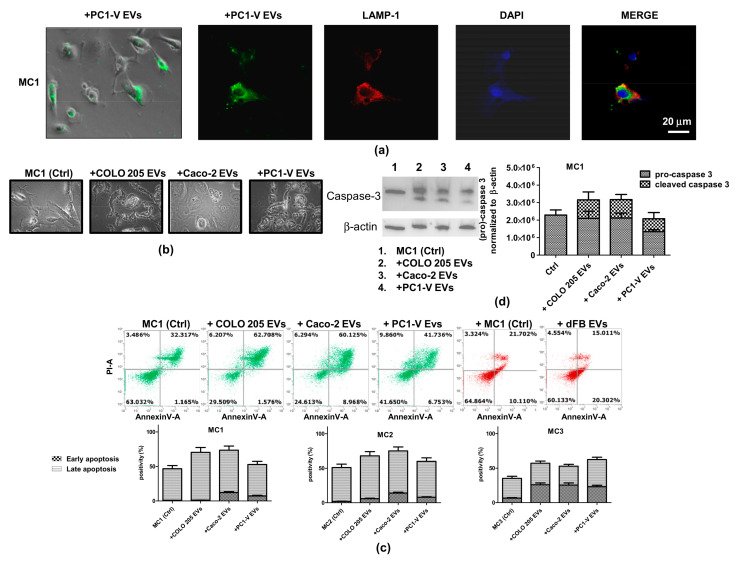
Uptake of tumor-EVs changes the morphology of mesothelial cells and causes their apoptosis. (**a**) Representative images of fluorescence and phase-contrast microscopy showing internalization of fluorescent PKH67-PC1-V EVs (green) in the MC1 cell line (magnification ×400) and representative images of fluorescence confocal microscopy showing PKH67-MC EVs uptake and colocalization with LAMP-1 (red) in PC1-V cells (magnification ×1000). (**b**) Representative images of optical microscopy showing the morphology of MC1 cells before (Ctrl) and after adding tumor EVs. (**c**) FCM dot plots of AnnexinV/PI positive MC1 cells before and after adding COLO 205 EVs, Caco-2-EVs, and PC1-V EVs and histogram plots depicting the % of early and late apoptosis. Each value reported in the histograms is the mean ± SD of three different experiments. FCM dot plots of AnnexinV/PI positive MC1 cells before and after adding dFB EVs are shown as the non-tumor model. (**d**) Representative immunoblots and quantification showing the increase in cleaved caspase-3 expression in MC1 cells treated with COLO 205 EVs, Caco-2 EVs, and PC1-V EVs. β-actin is used as a control.

Tumor-induced apoptosis of peritoneal MCs has been described as a mechanism that favors peritoneal invasion by gastric cancers [9]. Hence, by assuming an EV-mediated bidirectional crosstalk between CRC and mesothelial cells, we first characterized tumor EVs (Figure S7), as previously described for MC EVs, and went on to examine if they successfully entered mesothelial cells and caused apoptosis. The uptake of PC1-V EVs into MC1-2-3 cell lines was assessed by determining the colocalization of PKH67-stained green fluorescent-EVs and LAMP-1 in lysosomes and endosomes (Figure 4a and Figure S7). In order to investigate their proapoptotic potential, we added tumor EVs collected from COLO 205, Caco-2 and PC1-V to MC1, MC2, and MC3 cell lines and used FCM to analyze the morphological changes and apoptosis induced in the recipient cells. All three tumor EV populations disrupted mesothelial cell morphology, leading to an increase in abnormally shaped cells (Figure 4b). FCM analysis showed a considerable basal level of Annexin V/PI positive cells in all three MC cell lines, which was augmented by almost two-fold over the basal apoptosis level when Caco-2 and COLO 205 EVs were added, whereas PC1-V EVs slightly increased the Annexin V/PI positive cell fraction (Figure 4c). In order to confirm the involvement of tumor EVs in eliciting robust apoptosis, we performed the analysis by treating the MC1 cell line with isolated EVs from dermal fibroblasts (dFB) as non-tumor model. FCM results showed that EVs from dFB did not induce apoptosis (Figure 4c). Mechanistically, the tumor EVs caused increased expression and/or cleavage of pro-caspase-3 in MCs. This was seen in the MC1 cell line to which we added the Caco-2, COLO 205, and PC1-V EVs (Figure 4d, Figure S6b, and Table S3).

### 3.5. Tumor EVs Enhance MMT and Provoke a Senescent-Like State in Mesothelial Cells through the TGF-β Signaling Pathway, While Reducing Cellular Motility

**Figure 5:**
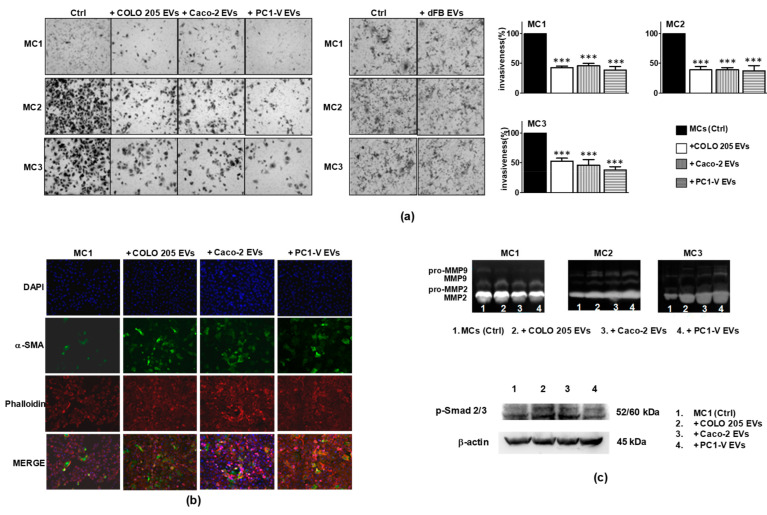
Tumor EVs enhance MMT and reduce the motility of mesothelial cells. (**a**) Representative images of optical microscopy showing reduction of MC1, MC2, and MC3 motility with COLO 205 EVs, Caco-2 EVs, and PC1-V EVs, MC1-3 invasiveness undisrupted by dFB EVs (magnification ×100) and quantification of mesothelial cell invasion % (*** *p* < 0.001 relative to control). Each value reported in the histograms is the mean ± SD of three different experiments performed in triplicate. (**b**) Representative images of fluorescence microscopy showing the increment of α-SMA (green) expression in phalloidin stained-MC1 (red) cells after the addition of COLO 205 EVs, Caco-2 EVs, and PC1-V EVs. Nuclei are counterstained with Dapi (blue) (magnification ×100). (**c**) Representative gelatin zymography showing that the addition of COLO 205 EVs, Caco-2 EVs, and PC1-V EVs to mesothelial cell lines increased the secretion of both pro-MMP-2/MMP-9 and active MMP-2/MMP-9, especially by MC2 and MC3 cell lines. Representative immunoblots showing the increase in p-Smad2/3 protein expression in MC1 treated with COLO 205 EVs, Caco-2 EVs, and PC1-V EVs. β-actin is used as loading control.

Tumor EVs actively participate in the reprogramming of MCs to facilitate tumor metastasis by inducing MMT [6,7,18]. It has also been demonstrated that peritoneal MCs are recruited to sustain CRC growth and progression toward the peritoneal cavity [15] and that a crucial event facilitating this process is the mesothelial-to-mesenchymal transition of MCs. To test the hypothesis that tumor EVs attract MCs by inducing MMT and cell motility, we performed migration and chemoinvasion experiments in which the MCs were layered onto Matrigel-coated porous filters and the tumor EVs were placed in the lower compartment of the well. We also tested the EVs collected from the CM of dFB on MC invasiveness to prove that the effect was selectively elicited by the tumor EVs. Morphological/molecular characterization of the MC lines showed that all three displayed a migration/invasion rate per se while the presence of tumor EVs strongly reduced MC invasiveness. dFB EVs did not modulate invasiveness (Figure 5a). In order to confirm the direct involvement of tumor EVs in strongly reducing the invasiveness of mesothelial cells, we performed the analysis by treating the MC1 cell line with EVs isolated from the CMs of PC1-V and dFB, with both the CMs deprived of EVs and the CMs as they are. In Figure S8, the results of such experiments showed that both the CM and the EVs obtained from PC1-V exerted a similar effect on MCs motility, instead the CM-EVs were almost ineffective, thus demonstrating the selective role of such EVs on MCs invasiveness. Accordingly, by performing the same assay, we found that the CMs and the EVs from dFB (used as the non-tumor model) did not affect MCs invasiveness. Nonetheless, addition of tumor EVs enhanced MMT in such cells, as demonstrated by the increase in α-SMA expression in MC1 (Figure 5b), by the release of active MMP-2/MMP-9, particularly evident in MC2 and MC3, and by the activation of Smad2/3 shown in MC1 in Figure 5c, Figures S3b and S2b, and Tables S3 and S4.

**Figure 6:**
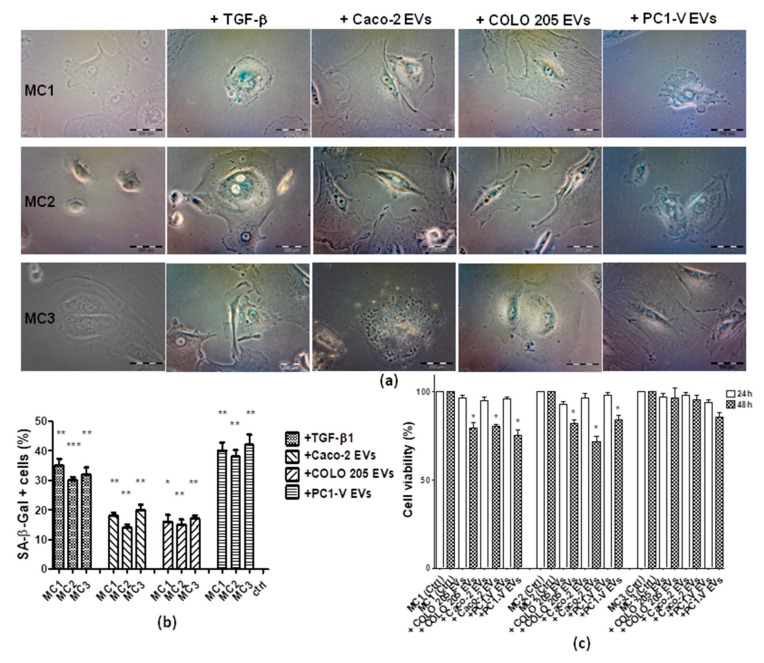
Senescent phenotype is induced by tumor EVs and TGF-β1 in MC cell lines. (**a**) Representative images of optical microscopy showing blue color staining for SA-β-Gal in MC1-3 especially after treatment with PC1-V EVs and TGF-β1. Treatment with COLO 205 EVs and Caco-2 EVs caused mainly abnormally shaped and apoptotic cells. (**b**) Histogram bars showing the percentage of SA-β-Gal positive cells. (*** *p* < 0.001, ** *p* < 0.01, * *p* < 0.05 vs. untreated cells) (**c**) Histogram bars showing the percentage of cell viability (* *p* < 0.05 vs. untreated cells).

Since a senescent state has been described as a cellular condition which transforms the protective function of MCs into peritoneal metastasis-supporting elements [12,13], we challenged the three MC lines with tumor EVs to ascertain if they led to senescence. After 24 h, the three MCs lines were assayed for a senescence-associated expression of β-galactosidase (SA-β-Gal) by optical examination. Interestingly, we observed phenotypic changes consistent with a senescent state mainly in the MCs treated with PC1-V EVs, whereas those treated with Caco-2 EVs and COLO 205 EVs mostly detached from the plate and showed modest SA-β-Gal staining. Assuming that a TGF-β feedback loop was involved in the crosstalk between MCs and PC1-V cells, and in order to gain insights into the bioactive molecule associated to PC1-V EVs, we treated the MC1-2-3 cell lines with TGF-β1 and found that it induced the onset of senescence, as evidenced by the SA-β-Gal staining of the cells (Figure 6a,b). Additionally, we tested the effect of tumor EV on MCs viability. We observed the reduction of cell viability by 48 h, particularly in MC1 and MC2 cell lines (Figure 6c), depending on EV origin. Collectively, these results indicated that acquisition of a senescent phenotype in MCs is dependent on tumor type.

### 3.6. EVs in the Peritoneal Lavage Fluid Facilitate Peritoneal CRC Dissemination

**Figure 7:**
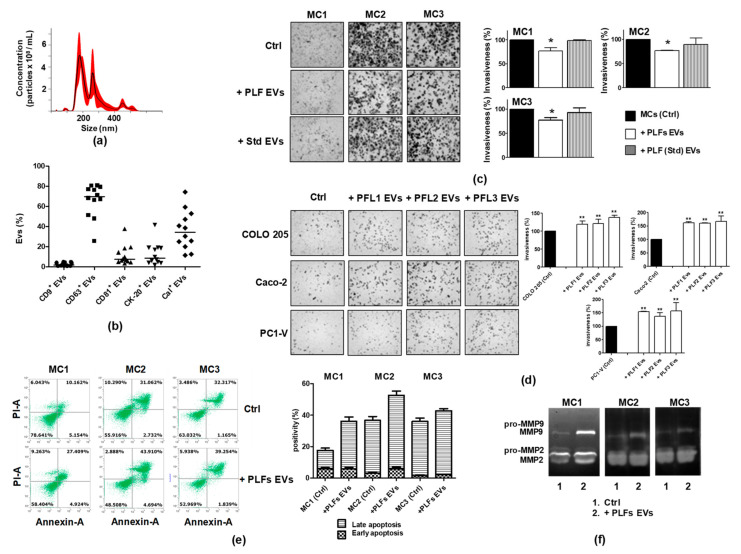
Biologically active EVs exist in the PLF and are engaged in establishing a pro-metastatic environment. (**a**) Representative nanoparticle tracking analyses showing the concentration and the size of EVs in the PLF. (**b**) FCM scatter plot with the median showing coexpression of CK-20 or Cal with the EV markers CD9, CD63, and CD81 in PLF EVs. (**c**) Representative images showing that a PLF EV pool isolated from a cancer patient reduces the motility of MC1, MC2, and MC3 cell lines, unlike the PLF EV pool from a non-tumor patient (Std) (magnification x100), and quantification of mesothelial cells invasion %. (**d**) Representative images showing that the PLF EV pool increases the motility of cancer cells and the quantification of tumor cells invasion %. Each value reported in the histograms (**c**,**d**) is the mean ± SD of three different experiments performed in triplicate (* *p* < 0.05 ** *p* < 0.01 relative to control). (**e**) FCM dot plots and quantification of AnnexinV/PI positive cells before and after applying a PLF EV pool to MC1-3, showing mainly induction of late apoptosis. (**f**) Representative gelatin zymography reveals that the addition of a PLF EV pool increases the secretion of active MMP-2/MMP-9 by MC1-3 cells.

To validate our results and demonstrate that the pool of EVs present in the PLF (PLF EVs) engages crosstalk between tumor cells and MCs and contributes to remodeling of the peritoneal environment, we investigated the effect of the EV pool on tumor cell and MC motility. As we did for MC EVs and tumor EVs, we preliminarily characterized the PLF EV pool. Representative images of this characterization are shown in Figure 7a,b. NTA and FCM were used to analyze the expression of three EVs-related tetraspanins, CD9, CD63, and CD81 [34], and of two cell biomarkers, CK-20 or calretinin, discriminating the amount of intestinal and MC-derived EVs present in the PLF EV pool, respectively [42,43].

Data analysis showed that the EVs were mainly positive for CD63 (median value: 69.82% vs. 2.11% and 7.61% for CD9^+^ and CD81^+^, respectively) and a large amount of them were released from mesothelial cells (median calretinin value: 34.63%), while only 8.67% were from colon cells (CK-20^+^) (Figure 7b). To evaluate the effects on MC motility, each of the 12 MC lines was layered onto the Matrigel-coated porous filters, while the EV pool isolated from the PLF of the same patient was placed in the lower compartment of the well.

As shown in Figure 7c, the PLF EV pools inhibited invasiveness of the MC1, MC2, and MC3 cell lines to varying degrees. Their effect was similar to and yet smaller than that of the tumor EVs.

The reliability of these findings was confirmed by conducting the same experiment with an EV pool isolated from the PLF of a non-tumor patient (EV Std). The motility of the MC cell lines remained almost unchanged as demonstrated by the data shown in Figure 7c. As reported for the MC1-3 lines, also the MC4-12 cell lines displayed a high migration/invasion rate per se while the presence of the PLF EV pool inhibited cell motility to a varying degree (Figure S9). Similarly, by testing the chemotactic effect of PLF EVs toward the COLO 205, Caco-2, and PC1-V cell lines, we observed an increase in tumor cell motility. We also tested the proapoptotic effect of the PLF EV pool on MC1, MC2, and MC3, and observed that the PLF EVs retained their ability to increase the basal level of apoptosis in these cells (Figure 7e) and cause the release of pro-MMP2/MMP9 and active MMP-2/MMP-9. Collectively, these results suggested that the PLF EV pool has the same chemokinetic potential towards tumor cells as observed in MC EVs as well as the capability to induce apoptosis and exacerbate the MMT of mesothelial cells (Figure 7f, Figure S3c, and Table S4).

### 3.7. Pantoprazole May Turn Out to Be a Useful Treatment to Reduce EV-Driven Tumor Metastasis

**Figure 8:**
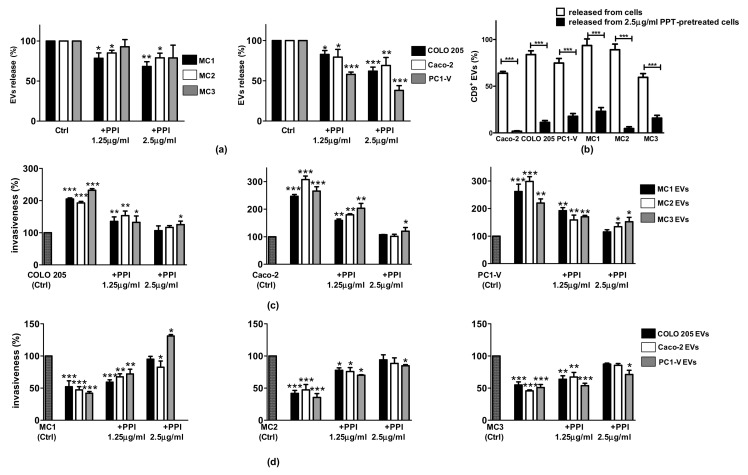
Pantoprazole is effective in reducing the release of EVs and their pro-tumorigenic activity. (**a**) Histogram bars reporting the percentage of EVs released by MC1-3 and cancer cell lines after treatment with 1.25 and 2.5 µg/mL pantoprazole (a PPI), showing a concentration-dependent reduction of EVs. (**b**) Histogram bars reporting the percentage of CD9^+^ EVs released by cancer and mesothelial cells after treatment with 2.5 µg/mL pantoprazole, showing a marked reduction of CD9^+^ EVs. (**c**) Histogram bars reporting cancer cell invasion percentage after treatment with EVs isolated from MCs treated with the PPI. (**d**) Histogram bars reporting MC invasion percentage after treatment with EVs isolated from cancer cells pretreated with the PPI. Data are shown as mean ± SD of three different runs. (* *p* < 0.05, ** *p* < 0.01, and *** *p* < 0.001 relative to untreated cells (Ctrl)).

Pantoprazole, a proton pump inhibitor (PPI) used in clinical practice and known to reduce the biogenesis of EVs [34], was used to demonstrate the crucial role of EVs in the crosstalk between tumor cells and MCs. To test the efficacy of pantoprazole in reducing the release of EVs from both cancer and mesothelial cell lines, we treated the cells with 1.25 and 2.5 µg/mL pantoprazole (similar to the plasma concentration [44]) and we then isolated the EVs from cell CMs and performed NTA and FCM analyses. Pantoprazole led to a concentration-dependent reduction of EVs released by MCs of about 10–30% (Figure 8a). Interestingly, the effect of pantoprazole was greater on the cancer cell lines. After treatment, Caco-2 released 20–40% fewer EVs, COLO 205 20–30% fewer, and the PC1-V cell line 46–65% fewer (Figure 8a). FCM showed a marked decrease only in the CD9^+^ EV population when they were released by cells pretreated with 2.5 μg/mL pantoprazole (Figure 8b). Next, we determined whether the EVs released after pantoprazole treatment still exerted biological effects. For this purpose, we carried out invasion assays with the same amount of EVs (200 µg/mL of protein EVs), purified by the conditioned medium of cancer or mesothelial cells treated with 1.25 and 2.5 µg/mL pantoprazole or left untreated. As shown in Figure 8b, the EVs released by 2.5 µg/mL pantoprazole-treated mesothelial cells were not very effective in increasing cancer cell line motility compared to the EVs released by untreated mesothelial cells, thereby demonstrating that treatment with pantoprazole strongly reduced the pro-tumor potential of MC EVs. Pantoprazole restored the motility of the mesothelial cell lines, thus changing the actions the EVs released by pantoprazole-treated cancer cells exerted on mesothelial cells (Figure 8c).

## 4. Discussion

The function of peritoneal MCs in CRC progression and peritoneal metastasis is still largely unknown and the tumor-induced transformation that helps them prepare the premetastatic environment in this disease is far from being elucidated. We investigated the tumor-promoting activity of peritoneal MCs from the PLFs of twelve CRC patients collected during surgery, assuming that they released EVs involved in the development of peritoneal metastasis. Peritoneal MCs derive from the exfoliation of the visceral peritoneum. As demonstrated by a large body of evidence, peritoneal MCs affected by tumor mediators present in the peritoneal cavity detach and drop off into the peritoneal fluids after undergoing MMT and apoptosis [9,43]. The PLF MCs examined displayed a significant apoptotic capacity and features consistent with MMT activation, such as massive release of pro-MMP2/MMP9 and active MMP2 and MMP-9, α-SMA expression, and a high migratory potential, all of which are distinctive of reactive mesothelial cells. The MCs also expressed high levels of the Homing-associated cell adhesion molecule (H-CAM/CD44) and released CD44^+^ EVs which reprogrammed the secretory profile of cancer cells by activating the β-catenin signaling pathway, causing upregulation of genes related to EMT and increasing cancer cell invasiveness. Of note, gene expression modulation by MC EVs was cell line specific, suggesting a tumor-dependent paracrine effect of MCs EVs. However, the final effect in all three CRC cell lines was to support cell mechanisms involved in the EMT of cancer cells and peritoneal metastasis [20,37,45]. Further evidence supporting the tumor-promoting role of the MC EVs was that tumor cells were stimulated to invade through a Matrigel-coated membrane under the chemotactic stimulus associated to the presence of MC EVs. This is consistent with recent findings demonstrating that, aside from metastatic cells travelling in blood stream, EV-mediated chemotaxis may play a pivotal role in the directional spread of cancer and suggests [39] that EVs may be of crucial importance for metastasis within body cavities such as the peritoneum. Further investigation into the role of EVs in the reciprocal interaction between cancer cells and MCs evidenced that all tumor EVs enhanced the MMT while reducing the invasion ability of MCs. This is in contrast with the findings reported by Gordillo et al. [15] who showed that increased MMT augmented mesothelial cell invasion. We observed a distinct effect by EVs from different tumors. Caco-2 EVs and COLO 205 EVs elicited a death-promoting effect in MCs, mainly by inducing apoptosis through caspase-3 cleavage and MMT. These findings are consistent with evidence that tumor cells damage mesothelial cells to breach the peritoneal barrier and facilitate peritoneal invasion [6]. PC1-V EVs instead, induced MMT, caused a slight increase in MC basal apoptosis, and predominantly triggered the onset of senescence in MCs, which points to a tumor-induced transformation of peritoneal MCs which confers to the latter a tumor-promoting role [13]. Considering that PC1-V are cells isolated from the ascites of a CRC patient with peritoneal carcinomatosis, it is conceivable that the EVs secreted by these cells are endowed with signals for reprogramming and enhancing the tumor-promoting role of mesothelial cells. TGF-β1 plays a crucial role in the activation of MMT and senescence of mesothelial cells [5,16,41]. We found that addition of exogenous recombinant TGF-β1 to MCs induced senescence in such cells basically to the same extent as caused by PC1-V EVs. However, when PC1-V EVs were added, we did not observe an increment of TGF-β1 protein expression in the recipient cells (data not shown) which nonetheless resulted in TGF-β signaling activation through the increase in p-smad2/3 expression in the mesothelial cells.

In order to understand if PLF EV analysis could directly predict prognosis and the risk of developing peritoneal metastasis in CRC patients, we isolated the EVs from the PLFs of 12 CRC patients. Despite some limitations in the design of the study due to the use of exogenous CRC cell lines, the PLF EV pool was added to the mesothelial cells from the PLF of each patient to perform ex vivo experiments. Analysis of the PLF EV pools showed that about 35% of the EV populations were released from mesothelial cells and almost 9% from colon cells. Although other cell types, such as stromal and immune cells, also release EVs in the peritoneal cavity, thereby influencing tumor–mesothelial cell crosstalk, our experiments demonstrated that the PLF EV pool elicited effects comparable to those induced by MC EVs and tumor EVs. As a proof of concept regarding how crucial it is to inhibit the trafficking of EVs, we used pantoprazole which reduced EV biogenesis and activity, showing that this treatment could prevent or reduce future metastasis. Interestingly, pantoprazole also reduced the expression of CD9 on EV surfaces. Collectively, our results suggest that PLF EVs may be a reliable tool to investigate the early signs of pathophysiological events in the peritoneal cavity and to identify potential prognosticators. This hypothesis has already been pursued by studying exosomal miRNAs from peritoneal lavage fluid as potential prognostic biomarkers of peritoneal metastasis in gastric cancer [46]. Recently, Roman-Canal. et al. [47] reported that EV-associated miRNAs can be utilized to characterize the biological and molecular characteristics of the CRC milieu, thus warranting further investigations into their role in colorectal peritoneal metastasis.

## 5. Conclusions

Although further studies are needed to identify the biomarkers in PLF EVs that may have a role as prognosticators of peritoneal carcinomatosis, our findings demonstrate that the PLF MCs of CRC patients are reactive cells, expressing CD44 on their membrane and releasing EVs that support remodeling of the peritoneal environment. We therefore suggest that peritoneal lavage cytology be performed and PLF EVs be utilized to integrate liquid biopsies and as pharmacological targets to improve the diagnosis and treatment of CRC patients in early stages of the disease.

## Figures and Tables

**Table 1:** List of primers used and their sequences.

GENE	Primer Sequences	
*uPA*	forward	5′-AGTGTCAGCAGCCCCACT-3′
	reverse	5′-CCCCCTGAGTCTCCCTGG-3′
*uPAR*	forward	5′-GCCCAATCCTGGAGCTTGA-3′
	reverse	5′-TCCCCTTGCAGCTGTAACACT-3′
*PAI1*	forward	5′-CTCCTGGTTCTGCCCAAGTT-3′
	reverse	5′-GAGAGGCTCTTGGTCTGAAAG-3′
*MMP-2*	forward	5′-AGCACCGCGACAAGAAGTAT-3′
	reverse	5′-ATTTGTTGCCCAGGAAAGTG-3′
*TGF-β1*	forward	5′-GACTACTACGCCAAGGAGGTCA-3′
	reverse	5′-TGCTGTGTGTACTCTGCTTGAAC-3′
*18S*	forward reverse	5′-CGGCTACCACATCCAAGGAA-3′5′-GCTGGAATTACCGCGGCT-3′

**Table 2:** Baseline characteristics of colon cancer patients.

Patient	Gender	Age	pSTAGING	T
Pz.1	M	57	T3 N1b (2/50) M0	T3
Pz.2	F	54	T4aN1a (1/37) M0	T4
Pz.3	F	71	T3 N0 (0/42) M0	T3
Pz.4	M	55	T4a N1b (2/32) M0	T4
Pz.5	M	78	T3 N0 (0/34) M0	T3
Pz.6	M	69	T4a N2b (12/21) M1a	T4
Pz.7	M	67	ypT3N1c M0	T3
Pz.8	M	60	T3 N0 (0/45) M0	T3
Pz.9	F	80	T3 N0 (0/32) M0	T3
Pz.10	F	81	T3 N2a (4/39) M0	T3
Pz.11	M	72	T2 N0 (0/11) M0	T2
Pz.12	M	41	T3 N0 (0/28) M0	T3

## Data Availability

The data regarding the EV characterization presented in this study are openly available in the EV-TRACK knowledgebase [48]. (EV-TRACK ID: EV210155).

## Supplementary Materials

The following are available online at https://www.mdpi.com/article/10.3390/cancers13102505/s1, Figure S1: Characterization of the peritoneal mesothelial cell lines, Figure S2: Whole blot related to Figure S1 and Figure 5c, Figure S3: Whole gel related to gelatin zymography assay, Figure S4: Establishment and characterization of primary cancer cell line PC1-V, Figure S5: PKH67-MC1 EVs are internalized in tumor cells and colocalize with lysosome marker LAMP-1, Figure S6: Whole blot related to Figure 2c and Figure 4d, Figure S7: Internalization and characterization of tumor-EVs, Figure S8: Modulation of MC1 invasiveness by CMs and EVs of PC1-V and dFB, Figure S9: PLF EVs pool reduces mesothelial cells motility, Table S1: Materials, Table S2: Ev isolation characteristics, Table S3: Intensity ratio of Western Blot images showing in Figures 2c, 4d, 5c, and densitometry readings and showing in S1c, Table S4: Densitometry readings of the gelatine zymography bands showing in Figure S3.
