# Supplementary material for: The Interaction between Reactive Peritoneal Mesothelial Cells and Tumor Cells via Extracellular Vesicles Facilitates Colorectal Cancer Dissemination
Source: Cancers (Basel). 2021 May 20;13(10):2505. doi: 10.3390/cancers13102505 (PMC8161093; doi:10.3390/cancers13102505)
Supplement: Supplementary file 1 [file cancers-13-02505-s001.zip › cancers-1208948-supplementary.pdf]

cancers

MDPI

Supplementary Material: The interaction between reactive peritoneal mesothelial cells and tumor cells via extracellular vesicles facilitates colorectal cancer dissemination

Simona Serratì, Letizia Porcelli, Francesco Fragassi, Marianna Garofoli, Roberta Di Fonte, Livia Fucci, Rosa Maria Iacobazzi, Antonio Palazzo, Francesca Margheri, Grazia Cristiani, Anna Albano, Raffaele De Luca, Donato Francesco Altomare , Michele Simone and Amalia Azzariti

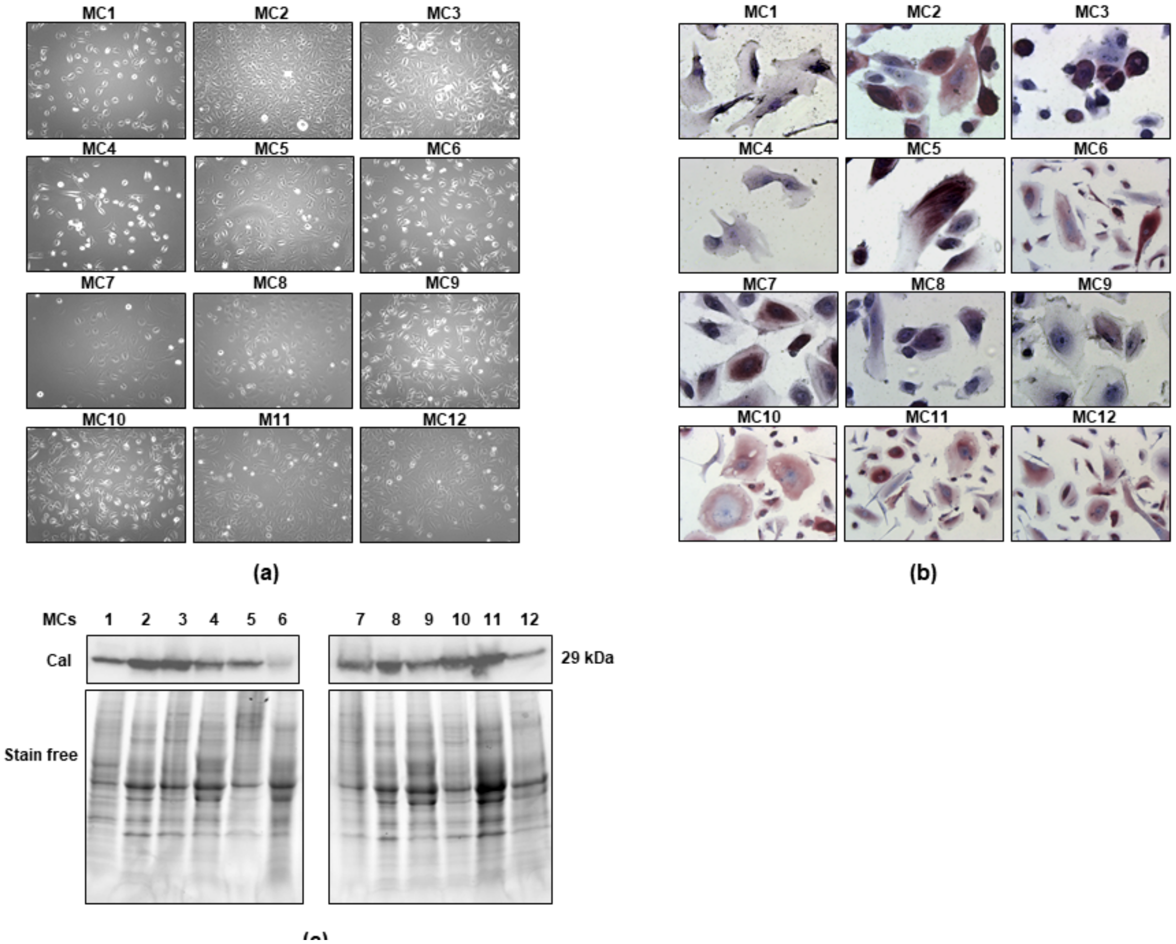

#

Figure S1. Characterization of the peritoneal mesothelial cell lines. **a**) Images of optical microscopy showing the morphology of the 12 MC cell lines isolated from the PLF of 12 colorectal cancer patients. **b**) Images showing the immunostaining of fixed MC cell lines with the anti-calretinin (Cal) antibody (red color) showing variable expression of the biomarker among the 12 cell lines. Counterstain of nuclei was done with Hematoxilin. (magnification x100). **c**) Western blot analysis of Cal protein expression in the 12 MC cell lines. Stain free detection iss used as loading control.

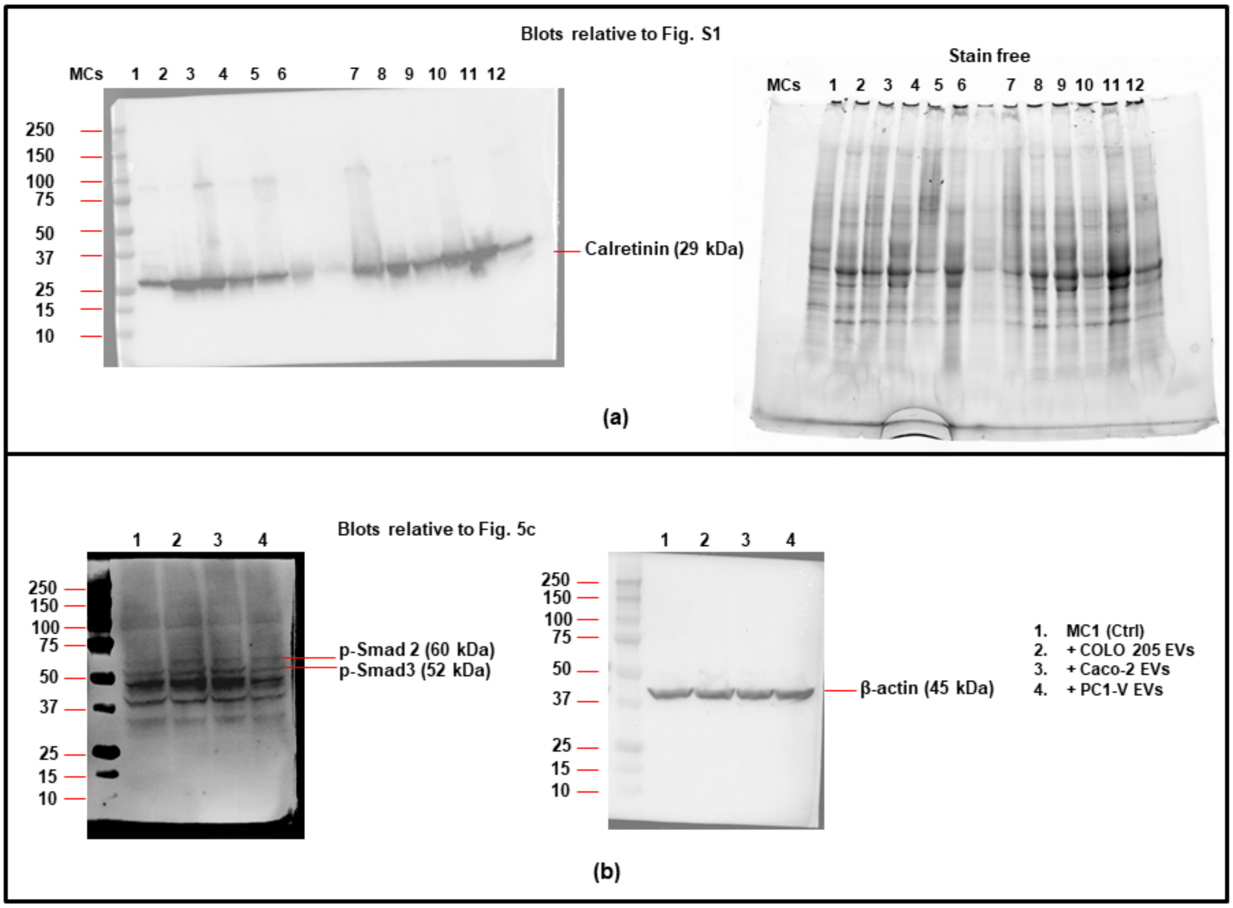

Figure S2. Whole blot related to Figure S1 and Figure 5c. Image**s** showing all the bands with all molecular weight markers on the Western Blot for Calretinin expression with relative complete stain-free gel (a), and whole blot for p-SMAD 2/3 expression with relative◎◎-Actin expression.

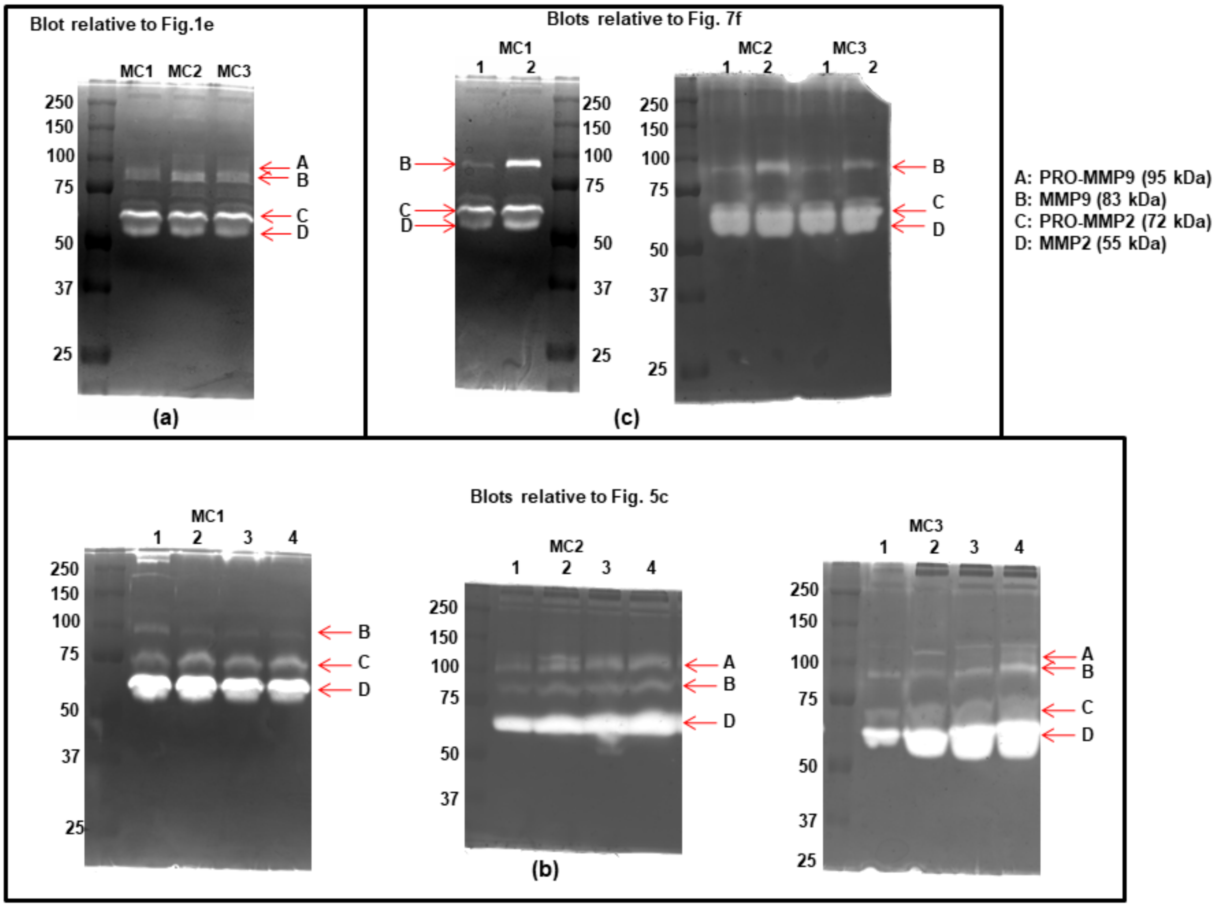

Figure S3. Whole gel related to gelatin zymography assay. Immages showing molecular weight markers relative to gelatin zymography rapresented in Figure 1 (a), Figure 5c (b) and Figure 7f (c).

Cell line	In vitro			In vivo				
	Growth pattern	Doubling time (h)	Cell morphology	sex	age	Original size of cell line	pathology	TNM stage
PC1-V	Adherent	34	Polygonal	M	42	Ascites	Peritoneal carcinosis from colon carcinoma	T4

(a)

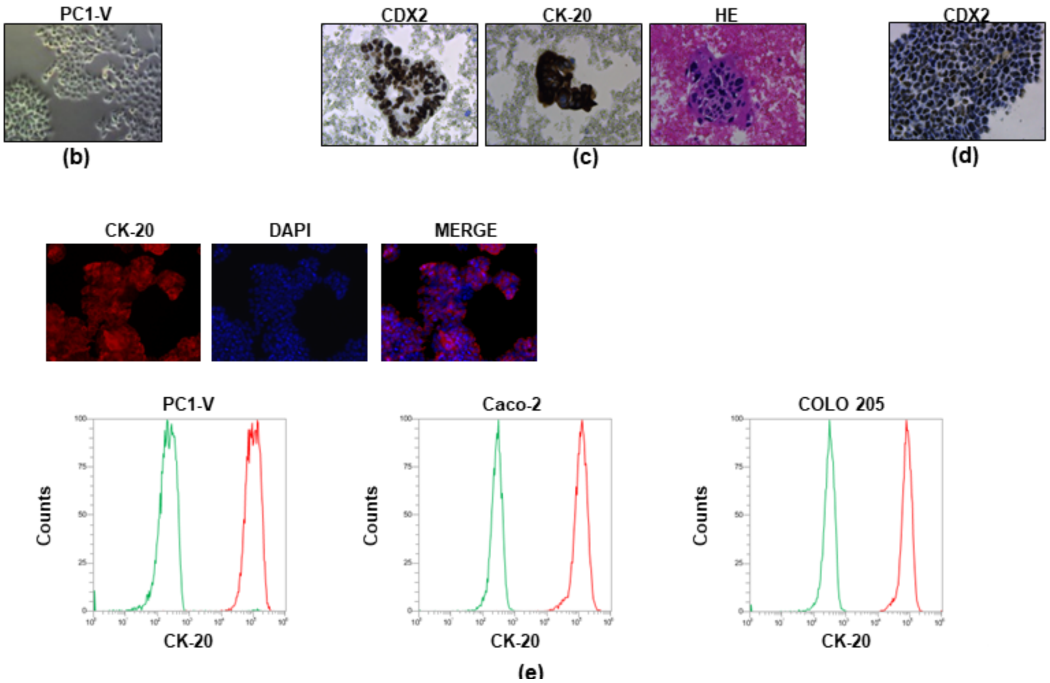

Figure S4. Establishment and characterization of primary cancer cell line PC1-V. **a**) In vitro and in vivo characteristics of PC1-V cells. **b**) Images of optical microscopy showing PC1-V cells displaying an epithelial-like morphology and an adherent growth. **c**) Images showing CDX2 and CK-20 positivity by immunoperoxidase staining and the HE staining of the patient cytological sample. D) CDX2 positivity of PC1-V cells by IHC. E) Images of fluorescence microscopy showing CK-20 positivity in PC1-V cells determined by IF, and FCM. The latter analysis shows the expression of CK-20 in comparison with COLO 205 and Caco-2 cell lines by FCS.

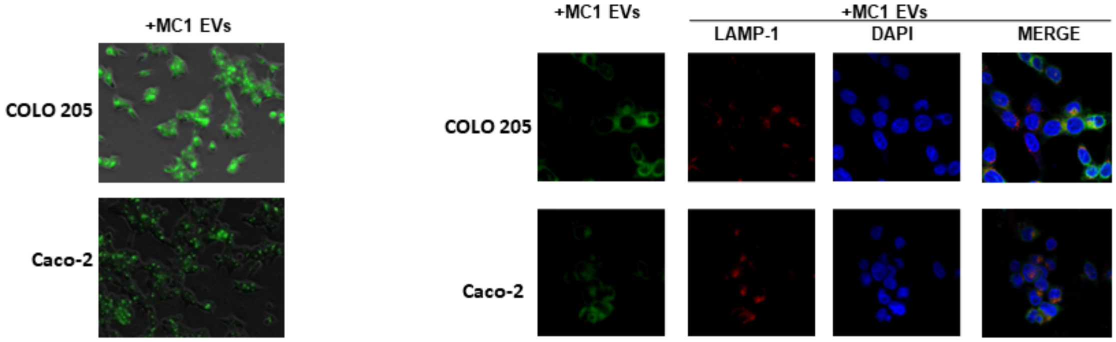

Figure S5. PKH67-MC1 EVs are internalized in tumor cells and colocalize with lysosome marker LAMP-1. Representative images of fluorescence and phase-contrast microscopy showing fluorescent PKH67-MC1 EVs (green) in Caco-2 and COLO 205 cell lines (magnification x400) and representative images of fluorescence confocal microscopy showing PKH67-MC1

EVs (green) uptake and colocalization with LAMP-1 (red) in Caco-2 and COLO 205 cell lines. Nuclei are counterstained with Dapi (blue) (magnification x100).

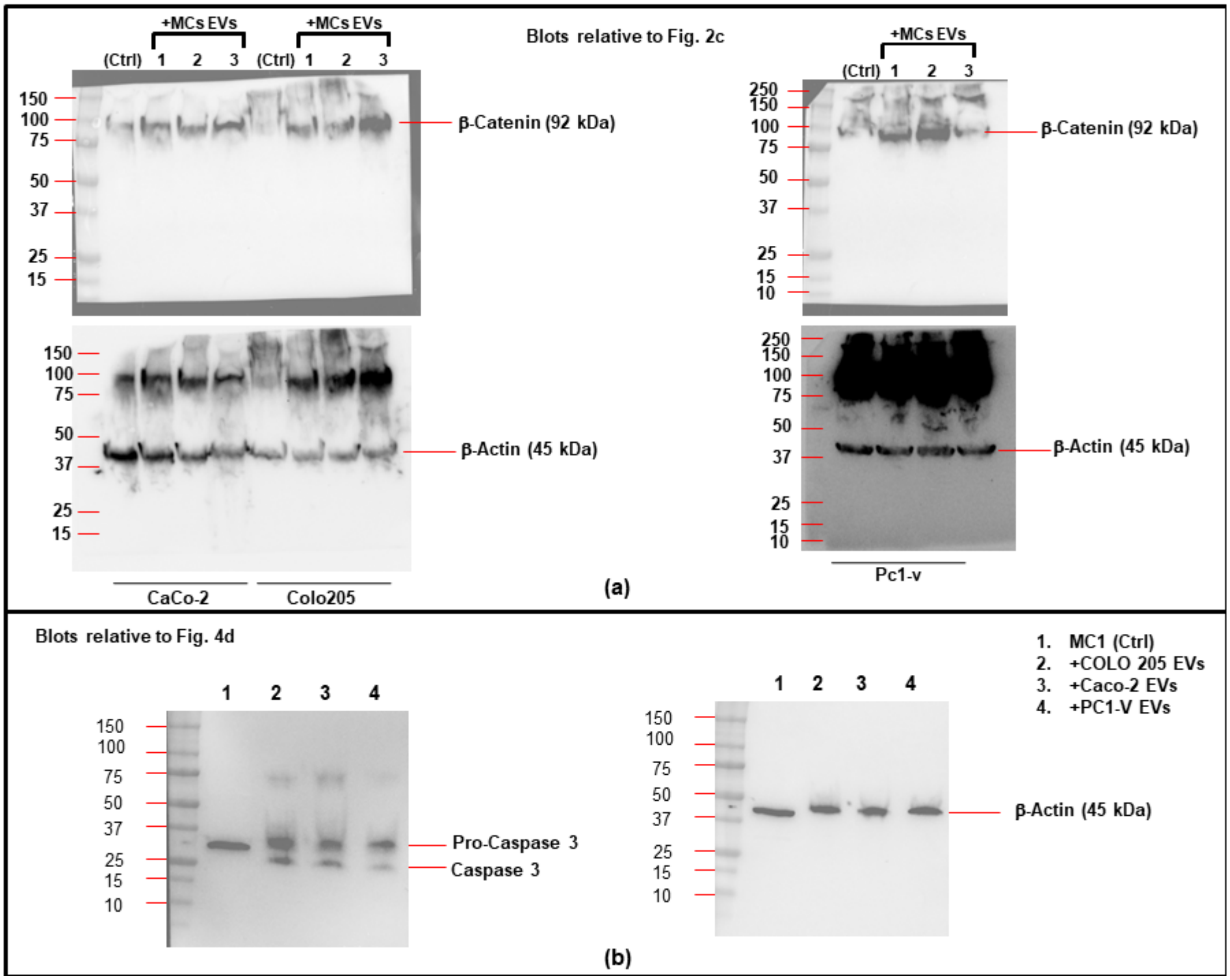

Figure S6. Whole blot related to Figure 2c and Figure 4d. Image**s** showing all the bands with all molecular weight markers on the Western Blot for ◎-Catenin with relative ◎-Actin expression (a), and whole blot for Caspase-3 expression with relative ◎-Actin expression (**b**).

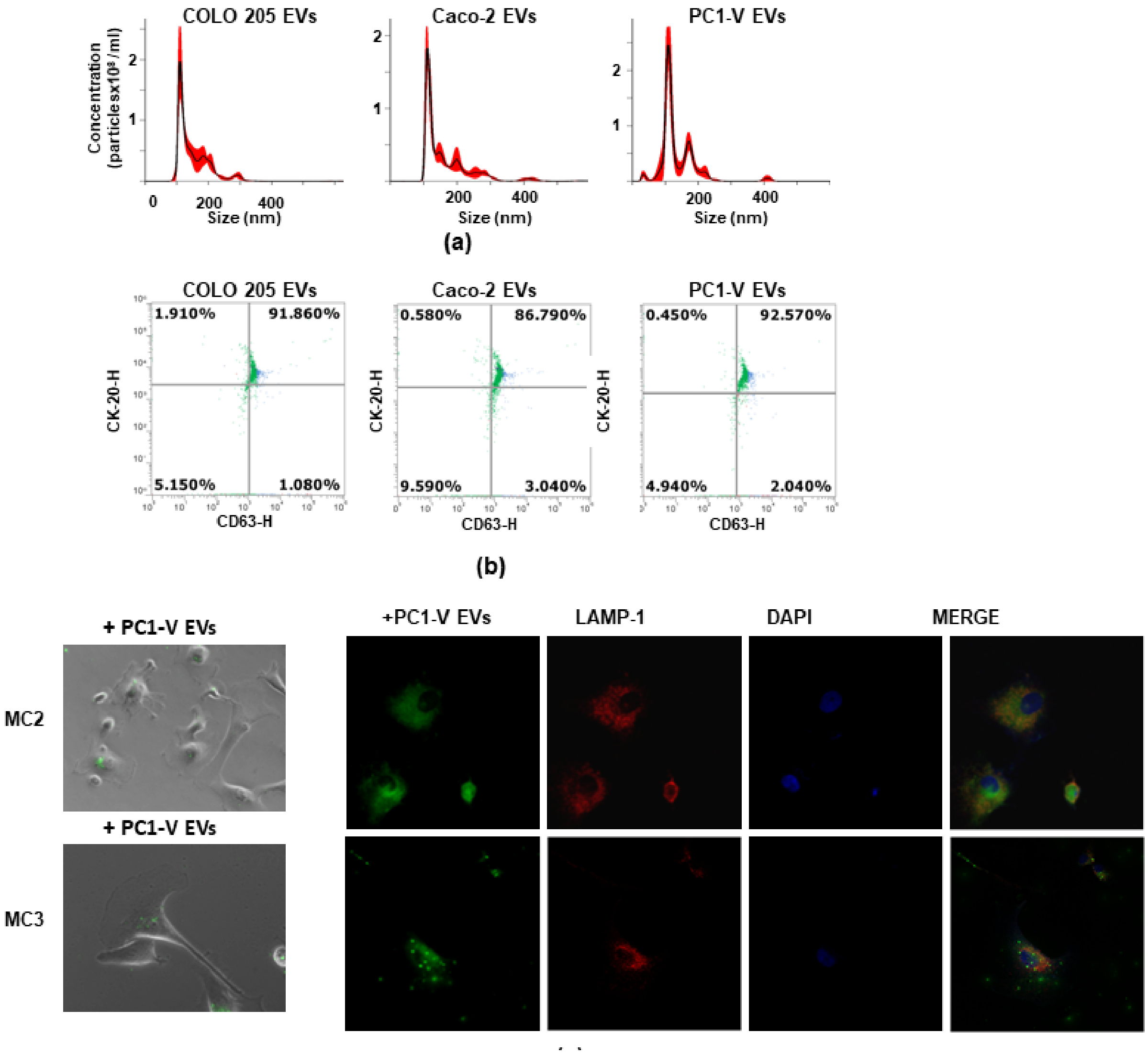

Figure S7. Internalization and characterization of tumor-EVs. **a)** Nanoparticle tracking analyses showing the concentration and the size of EVs released by tumor cell lines. **b**) Dot plots corresponding to the FCM analysis of EVs released by tumor cells (CK-20), showing that the majority of them are CD63$^+$ one. **c**) Representative images of fluorescence and phase-contrast microscopy showing fluorescent PKH67-PC1-V EVs (green) in MC2 and MC3 cell lines (magnification x400) and representative images of fluorescence confocal microscopy showing PKH67-PC1-V EVs (green) uptake and colocalization with LAMP-1 (red) in Caco-2 and COLO 205 cell lines. Nuclei are counterstained with Dapi (blue) (magnification x100).

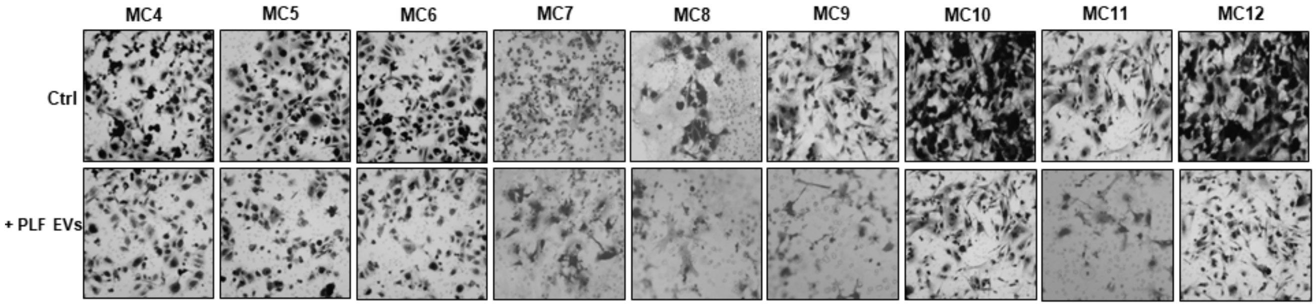

Figure S8. PLF EVs pool reduces mesothelial cells motility. Representative images of optical microscopy showing that PLF EVs pool isolated from the PLF of each patient reduces the motility of MC4-12 cell lines.

Table S1. Materials. Flow Cytometry (FCM), Immunohistochemistry (IHC), Immunofluorescence (IF), Western blot (WB).

Product	Catalog n.	Company	Assay	Concentration of usage
anti-CD9-Super Bright 436	62-0098-42	e-Biosciences, Thermo Fisher Scientific, Waltham, MA USA	FCM	5 µL (0.25 µg)/test
anti-CD63-PE-Cyanine7	25-0639-42	e-Biosciences, Thermo Fisher Scientific, Waltham, MA USA	FCM	5 µL (0.25 µg)/test
anti-CD81- PerCP-eFluor 710	44-0819-42	e-Biosciences, Thermo Fisher Scientific, Waltham, MA USA	FCM	5 µL (0.25 µg)/test
anti-CD44-VioBlue	130-113-337	Miltenyi Biotec, Bergisch Gladbach, Germany	FCM	2 µL /test
anti-CK-20-PE	ab209923	Abcam, Cambridge, UK	FCM	1/500
anti-a-SMA	A5228	Sigma-Aldrich, St. Louis, MO, USA	IF	2 µg /ml
anti-Vimentin	V6630	Sigma-Aldrich, St. Louis, MO, USA	IF	1:500
anti-Lamp-1	#9091	Cell Signaling Technology, Danvers, MA, USA	IF	1:200
anti-β-Actin	#3700	Cell Signaling Technology, Danvers, MA, USA	WB	1:1000
anti-◎-Catenin	#8480	Cell Signaling Technology, Danvers, MA, USA	WB	1:1000
anti- p-Smad2/3	#8828	Cell Signaling Technology, Danvers, MA, USA	WB	1:1000
anti-CK-20	MA5-31979	Invitrogen, ThermoFisher Scientific, Waltham, MA USA	WB, IHC	1:1000, 1:200
anti-Calretinin	PA5-16681	Invitrogen, ThermoFisher Scientific, Waltham, MA USA	WB	1:500
anti-uPAR (R-4)	MON-R-4-02	Invitrogen, ThermoFisher Scientific, Waltham, MA USA	WB	1:500
anti-CDX2	PA0375	Novocastra, Leica Biosystems, Wetzlar, Germany	IHC	According the manufacturer instructions.
anti-Caspase 3	sc-7272	Santa Cruz Biotechnology, Inc	WB	1:500
Alexa Fluor™ 568 goat anti-mouse IgG	A11004	Molecular Probes, Life Thecnologies, ThermoFisher Scientific, Waltham, MA USA	IF	1:1000
Alexa Fluor™ 488, Goat anti-Mouse IgG	A11001	Molecular Probes, Life Thecnologies, ThermoFisher Scientific, Waltham, MA USA	IF	1:1000
Goat Anti-Mouse IgG (H + L)-HRP Conjugate	#1706516	BIORAD, Hercules, CA, USA	WB	1:2000
Goat Anti-Rabbit IgG (H + L)-HRP Conjugate	#1706515	BIORAD, Hercules, CA, USA	WB	1:2000
**Other materials**				
SB complete staining buffer	SB-4401-42	e-Biosciences, Thermo Fisher Scientific, Waltham, MA USA	FCS	2 µL /test
Phalloidin-iFluor 555	ab176756	Abcam, Cambridge, UK	IF	1:500

DAPI (4',6-Diamidino-2-Phenylindole, Dihydrochloride)	D1306	Invitrogen, ThermoFisher Scientific, Waltham, MA USA	IF	300 nM
Green Fluorescent Cell Linker	MIDI67-1KT	Sigma-Aldrich St. Louis, MO, USA	IF	According the manufacturer instructions.
FITC Annexin V Apoptosis Detection Kit I	556547	BD Pharmingen™, San Jose, CA, USA	FCM	According the manufacturer instructions.
Pantoprazolo	AIC n. 044465019	SUN Pharma, Sun Pharmaceutical Industries Ltd. Goregaon, Mumbai, India	Functional studies.	1.25 and 2.5 µg/mL

Table S2. Intensity ratio of Western Blot images showing in Figures 2c, 4d, 5c, and densitometry readings and showing in S1c.

**Fig. 2c**	**⚜-catenin normalized to ⚜-actin**			
	**Ctrl**	**1**	**2**	**3**
**CaCo-2**	2758321	7242274	8653667	16913705
**Colo205**	1832862	12729893	12410528	28636569
**PC1-V**	3694842	6462198	12200514	3502585
**Fig. 4d**	**Caspase-3 normalized to stain free loading sample**			
**MC1**	**1**	**2**	**3**	**4**
**Pro-caspase 3**	2746633,0	2873901,0	2671387,0	1406835,0
**Pro-caspase 3**	2364687,0	1926893,0	1724544,0	1116577,0
**Pro-caspase 3**	1746053,0	1497555,0	1945360,0	1482869,0
**Cleaved-caspase 3**	0,0	1950548,0	1628676,0	1457912,0
**Cleaved-caspase 3**	0,0	705569,0	670084,0	350212,0
**Cleaved-caspase 3**	0,0	510012,0	860598,0	399062,0
**Fig. 5c**	**p-Smad 2/3 normalized to ⚜-actin**			
	**1**	**2**	**3**	**4**
**MC1**	650268	1010550	1034423	601284
**Fig. S1c**	**Calretinin normalized to stain free loading sample**			
**MC1**	29929760			
**MC2**	46105576			
**MC3**	30430095			
**MC4**	36015056			
**MC5**	23936169			
**MC6**	20235378			
**MC7**	62560320			
**MC8**	45822171			
**MC9**	36938573			
**MC10**	39728671			
**MC11**	35155302			
**MC12**	18000704			

Table S3. Densitometry readings of the gelatine zymography bands showing in Figure S3.

	Volume (Int)			
**Fig. 1e**	***MC1***	***MC2***	***MC3***	
PRO-MMP9	19562810	29291036	24691106	
MMP9	16427924	24536384	18150410	
PRO-MMP2	177001112	155704902	158612306	
MMP2	118238424	101007572	86408920	
**Fig. 5c**	***MC1 - 1***	***MC1 - 2***	***MC1 - 3***	***MC1 - 4***
MMP9	24391872	8686700	9257616	6643020
PRO-MMP2	46332176	85406320	75051604	93743856
MMP2	708335036	713850852	591925624	571421832
**Fig. 5c**	***MC2 - 1***	***MC2 - 2***	***MC2 - 3***	***MC2 - 4***
MMP9	22999633	55709908	76030263	35807966
PRO-MMP2	18996963	46943491	48648793	61235576
MMP2	403678870	621366515	742713670	787844249
**Fig. 5c**	***MC3 - 1***	***MC3 - 2***	***MC3 - 3***	***MC3 - 4***
PRO-MMP9	807618	2615412	1454637	3783423
MMP9	25042791	22703754	35798502	79788759
PRO-MMP2	19690563	49007016	76343619	70492308
MMP2	241223718	579251850	701774817	737940345
**Fig. 7f**	***MC1 - 1***	***MC1 - 2***		
MMP9	13349760	130981260		
PRO-MMP2	138386052	176989788		
MMP2	70971804	147619704		
**Fig. 7f**	***MC2 - 1***	***MC2 - 2***	***MC3 - 1***	***MC3 - 2***
MMP9	74021850	150233370	49366590	52628520
PRO-MMP2	269053260	303433830	270572610	294616350
MMP2	199226790	218666910	188038200	199434270
